# Numb Chin Syndrome as First Symptom of Diffuse Large B-Cell Lymphoma

**DOI:** 10.1155/2014/413162

**Published:** 2014-12-16

**Authors:** Mario Carbone, Francesco Della Ferrera, Lucio Carbone, Gaia Gatti, Marco Carrozzo

**Affiliations:** ^1^Department of Surgical Sciences, Oral Medicine Section, CIR Dental School, University of Turin, Via Nizza 230, 10126 Turin, Italy; ^2^Private Practice, Turin, Italy; ^3^Department of Medical Sciences, Section of Pathology, San Giovanni Battista Hospital, Strada Comunale di S. Vito Revigliasco 34, 10133 Torino, Italy; ^4^Oral Medicine Department, Centre for Oral Health Research, Newcastle University, Newcastle upon Tyne, Tyne and Wear NE1 7RU, UK

## Abstract

Numb chin syndrome is a rare sensory neuropathy of the mental nerve characterized by numbness, hypoesthesia, paraesthesia, and very rarely pain. Dental causes, especially iatrogenic ones, maxillofacial trauma, or malignant neoplasm are etiologic factors for this rare syndrome. Many malignant and metastatic neoplasms are causing this syndrome, like primary osteosarcoma, squamous cell carcinoma, and mandibular metastasis of primary carcinoma of breast, lung, thyroid, kidney, prostate, and nasopharynx. Haematological malignancies like acute lymphocytic leukaemia, Hodgkin and non-Hodgkin lymphoma, and myeloma can cause this neuropathy. The authors report a case of a 71-year-old woman in which the numb chin syndrome was the first symptom of the diffuse large B-cell lymphoma, which caused infiltration and reabsorption of the alveolar ridge and lower mandibular cortex. A biopsy of the mass was performed on fragments of tissue collected from the mandibular periosteum, medullary and cortical mandibular bone, and inferior alveolar nerve.

## 1. Introduction

Numb chin syndrome (NCS) or mental nerve neuropathy is a rare sensory neuropathy characterized by numbness, hypoesthesia, paraesthesia, and very rarely pain in the distribution of the mental nerve. There are many etiologic factors for this rare syndrome: dental causes, especially iatrogenic ones (oral surgery), are by far the most common. If not related to a dental cause, this innocuous complaint is considered a “red flag” symptom of a malignant neoplasm. In this case NCS can be the first symptom of cancer but more often is the sign of its relapse or progression in a patient with malignancy history [1]. Malignant and metastatic neoplasms are more commonly causing this syndrome. However, primary tumours like primary osteosarcoma of mandible and squamous cell carcinoma can be responsible for NCS. Among malignant distant neoplasms that metastasize to the mandible, the most frequent is breast cancer followed by primary carcinoma of lung, thyroid, kidney, prostate, and nasopharynx. Other associated neoplasms include hematological malignancies like acute lymphocytic leukemia, Hodgkin and non-Hodgkin lymphoma (NHL), and myeloma. Tumors of the inferior alveolar nerves and mental nerves and their sheaths as well as the compression of the mandibular division of trigeminal nerve at the base of the skull by a tumor mass or leptomeningeal invasion may also cause NCS. Trauma (fractures of ramus or body of the mandible) and systemic diseases such as sickle cell anaemia, multiple sclerosis, amyloidosis, sarcoidosis, and diabetes mellitus can be responsible for this unusual symptom as well [2].

NHL is a particular type of lymphoma in which malignant neoplastic proliferation of lymphocytes at different stages of mutation occurs. Nearly 40% of NHL arises in extranodal sites and the head and neck region is the second most frequent anatomic site of extranodal NHLs. Half of the extranodal NHLs of the head and neck are located in the Waldeyer ring. Extranodal lymphomas arising in the oral cavity account for less than 5% of all oral malignant neoplasms and they represent the third most common neoplasm involving oral cavity following squamous cell carcinoma and salivary glands neoplasm. Incidence and mortality from NHL have risen since 1970 in the developed countries and now NHL is still the sixth most common cause for cancer-related deaths in the USA [3].

However recent epidemiologic studies found that since 1990 the incidence and mortality from NHL seem to level off in Europe as in other developed areas of the world [4].

The histologic types of NHLs commonly found in the head and neck are B-cell neoplasms. Within these types the diffuse large B-cell lymphoma (DLBCL) and extranodal marginal zone lymphoma of the mucosa associated lymphoid tissue (MALT) are the most frequent. DLBCL often involves the Waldeyer ring but can be also found in the soft tissue and bone of the jaws [3].

In past years many studies investigated the association between chronic inflammatory diseases such as rheumatoid arthritis, Sjogren's syndrome, and the development of lymphoma. Optimal treatment has not yet been established and this lymphoma is not infrequently associated with fatal outcome [5].

We present a case in which the cause of NCS was a DLBCL of the mandible.

## 2. Case Report

A 71-year-old Caucasian woman was referred to our department in September 2013 because of the development of anaesthesia of the lower right lip and chin. This symptom was preceded by a sudden diffuse pain in the right mandible which lasted a few hours one month before the development of the neurological impairment. A dental extraction in the anterior region of the mandible was performed by dental practitioner due to severe periodontal disease after the onset of the first sudden mandibular pain. History taking of the patient revealed that she suffered from hypertension, chronic obstructive pulmonary disease, rheumatoid arthritis (RA), and an IgM monoclonal gammopathy of undetermined significance (IgM-MGUS). At the first clinical evaluation she was taking methotrexate (MTX) (10 mg once a week), folic acid (5 mg/daily), prednisone (7.5 mg/daily), ibuprofen (80 mg/daily), and calcium.

The intraoral clinical examination was unremarkable, but an ulcer of 1 cm wide could be seen in the lower lip. A painful hard swelling was evident on palpation in the lower right vestibular fornix in the premolar area and in homolateral submandibular space. Lymphadenopathies were detected in the right later cervical, supraclavicular, and subaxillary groups and in the left axillary group. The patient suffered from chronic periodontitis which during years determined tooth loss and prosthetic therapy with dental implants and partial removable dentures. There were no direct dental or other local causes which could explain the onset of anaesthesia of right inferior alveolar nerve.

**Figure 1:**
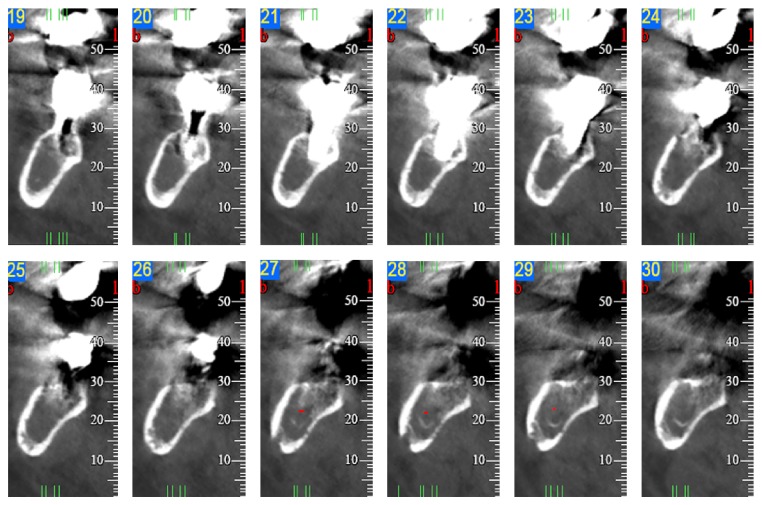
CT-scan examination. Note microlacunar reabsorptions of the mandibular cortices and hypodensity of the spongiosa, diffused from 4.5 to 4.8.

A dental panoramic tomography (DPT), a maxillofacial CT scan, and an ultrasound (U/S) of the neck were performed to assess the morphology and the limits of the lesion. The DPT revealed a radiopaque area diffused from 4.5 to 4.8 with some microlacunae on the alveolar ridge and lower mandibular cortex. The CT scan confirmed the microlacunar reabsorptions due to bony structural rearrangement of all the mandibular cortices peripheral to the radiopaque lesion diffused from 4.5 to 4.8. The centre of the lesion was characterized by hypodensity of the spongiosa. The mandibular canal was detectable only in the distal sections (Figure 1). In the U/S, a 2 cm wide hypoechogenic mass was clearly visible. This mass was very close to the right corpus of the mandible with well-defined margins. The cortical bone peripheral to this finding revealed signs of cortical erosion. Three swollen lymph nodes were detectable close to the mass in the submandibular space. These glands showed a metastatic pattern. Other reactive lymph nodes were detectable bilaterally in the later cervical groups.

**Figure 2:**
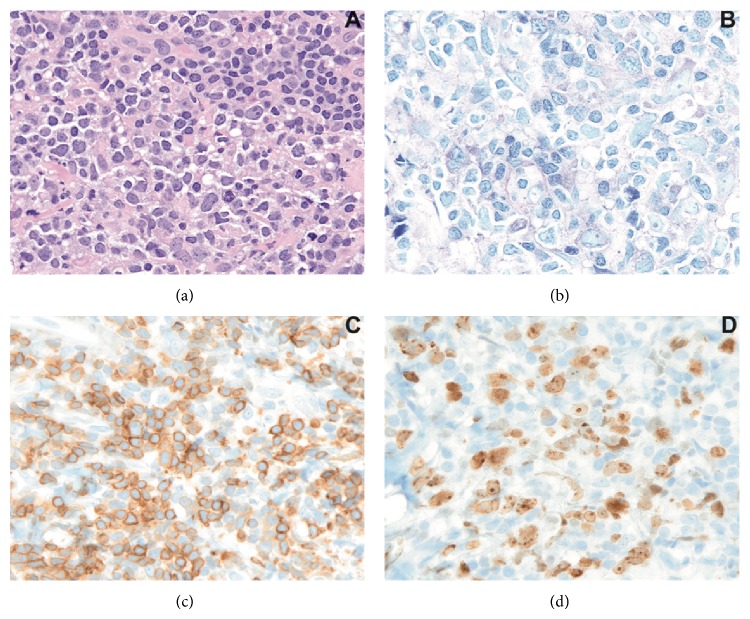
(a)-(b) Histopathological examination (hematoxylin-eosin and Giemsa stains); (c) immunohistochemical reactions for BCL2+; and (d) proliferation index (Ki67, MIB1). Magnification: 400x.

The patient was then referred to the maxillofacial surgery department in order to proceed with a biopsy of the mass and obtain a histopathology characterization of the lesion. Fragments of tissue were collected from the mandibular periosteum, medullary and cortical mandibular bone, and inferior alveolar nerve. The histopathological examination of the material obtained with the biopsies demonstrated a diffuse proliferation of large lymphoid cells with quite abundant basophil cytoplasm and pale perinuclear ring, oval nuclei with dispersed chromatin, and one or more nucleoli (Figure 2, hematoxylin-eosin 400x). Immunohistochemical reactions were performed and the neoplastic cells were diffusely positive for CD20 and BCL2 and weakly positive for BCL6. The proliferation index, evaluated with Ki67, MIB1 clone, was high: about 70%.

After the diagnosis of DLBCL the patient underwent a PET with 18-FDG and a total body CT-scan with contrast dye for proper staging of the neoplasm. The PET examination revealed an increased uptake in right mandible and in lymph nodes of the supradiaphragmatic, mediastina, aortic, and subcarinal groups. Other lymphadenopathies were detected by the CT-scan bilaterally in the submandibular, digastric groups and in the right subclavicular group.

The final diagnosis revealed a DLBCL IVA with bone marrow involvement (BM+) and a monoclonal IgM component (CM IgM-MGUS) in association with RA.

The patient was then referred to the department of haematology to initiate proper treatment.

## 3. Discussion

In our case the onset of NCS represented the first specific symptom of a hematologic malignant neoplasm. It has been calculated that NCS could be the inaugural manifestation of a malignancy in 30% of cases and the first sign of relapse or progression of cancer in patients with malignancy history in 40% of cases [6]. The most common neoplastic cause of NCS is metastatic breast cancer (40% cases) followed by NHL (20%) and prostate cancer (6%) [6].

The particularity of the present case includes the onset of DLBCL in association with RA, IgM-MGUS, and the previous therapy with methotrexate for RA. Several studies have linked certain autoimmune and chronic inflammatory conditions, including rheumatoid arthritis (RA), Sjogren's syndrome, systemic lupus erythematosus (SLE), celiac disease, and chronic thyroiditis, to an increased risk of lymphoma. The lymphoma risk in RA ranged from 1.5 to 4 [7]. More recent studies reported a 2-fold increase in risk. It has been suggested that considerable heterogeneity in risk among subsets of RA patients does probably exist. The risk is likely higher in patients with the most severe disease and lower in those with mild to moderate disease. Much of the increased lymphoma risk in RA patients is due to factors directly related to the disease or its treatment. The inflammatory activity seems to be a driving force in lymphoma development, but the exact pathogenesis still remains unclear. The influence of treatment with disease modifying antirheumatic drugs, including methotrexate, azathioprine, and other immunosuppressive substances, has also been investigated. Notably, in the present case, MTX was used to control RA and this drug has been associated with often EBV positive lymphoproliferative disorders in several case reports [7]. However recent studies found that there is little evidence that methotrexate alone (as used to treat inflammatory disorders) increases lymphoma risk [8].

Another particular feature of our case was the presence of an IgM monoclonal gammopathy. This disorder is characterized by a homogeneous immunoglobulin (the M-protein) spike in serum or urine. MGUS is per se a benign pathology which is differentiated by multiple myeloma, Waldenström's macroglobulinemia (WM), and NHLs according to the type of M-protein, its concentration, degree of bone marrow infiltration of plasma cells or lymphoplasmacytic cells, and presence of certain clinical manifestations. The available data suggest that MGUS is present in ~3% of the general white population ≥50 years of age and is predominantly incidentally diagnosed. The majority of MGUS patients do not progress to a lymphoproliferative disorder. The average risk of progression is estimated to be 1%/yr, and the 25-year cumulative risk is 30% [9].

It is also interesting that MGUS as other monoclonal gammopathies has been associated with neuropathies. However this type of peripheral neuropathy does not usually involve the cranial nerves [10].

The exact pathophysiology of NCS in patients with cancer is still unknown. Metastasis to the mandible and bone marrow infiltration of the jaw leading to compression of the nerve may be important mechanisms in hematologic cancers and breast cancer. Direct perineural and neural invasion may be an important pathway in primary tumours of the inferior alveolar nerve, squamous cell carcinoma, and lymphoma. It has been described that malignancies in their initial stage are accompanied by a significant inflammatory response. This response can be responsible for prolonged oedema and inflammation. The possible confinement of the inflammatory process within the bony canal may lead to ischemia and nerve damage [1].

NCS's diagnosis is essentially clinical; however, various radiographic studies are helpful to confirm diagnosis especially when a neoplastic origin is suspected. The first step exam could be the DPT which provides a significant image of the maxillofacial skeleton and dentition. The major limits of this technique lay in its lack of fine definition of the image and in its bidimensionality. These limits could be overcome with complementary examinations like intraoral radiographies and CT-scan extended to the brain and the base of the skull in order to exclude intracranial origin of NCS. In addition to those radiographic studies MRI and PET could be useful to obtain an increased level of anatomic details and to show disease distribution through metabolic cell activity.
